# 2218. Evaluation of Bacteremia in Febrile Neutropenia and Implications on Antimicrobial Stewardship

**DOI:** 10.1093/ofid/ofad500.1840

**Published:** 2023-11-27

**Authors:** Sowmya Kalava, Niki Arab, Rolando Zamora Gonzalez, Brian Kim, Arthur Jeng

**Affiliations:** University of California Los Angeles, Los Angeles, California; Olive View- UCLA Medical Center, Los Angeles, California; University of California Los Angeles, Los Angeles, California; Olive View-UCLA Medical Center, Sylmar, California; Olive View UCLA Medical Center/UCLA School of Medicine, Sylmar, California

## Abstract

**Background:**

Febrile neutropenia (FN) is a common complication in cancer patients. IDSA guidelines for FN in cancer patients recommend the initiation of an empiric antibiotic (abx) against *Pseudomonas aeruginosa* (PsA). At Olive View-UCLA Medical Center (Sylmar, CA), we reviewed FN cases to determine the prevalence of PsA bacteremia and assess the necessity of anti-PsA abx.

**Methods:**

Retrospective chart review on all adult (≥ 18 years) positive blood cultures (Bcx) from January 1, 2017 to December 30, 2022. Positive Bcx were matched with ICD-10 code for neutropenia on inpatient encounter. Exclusion criteria consisted of absolute neutrophil count ≥ 500, FN not related to malignancy, and untreated suspected contamination. Charts were reviewed for Bcx results, source of bacteremia, antibiotic prescribing, and 30-day all-cause mortality.

**Results:**

Of 1,303 neutropenia encounters and 3,755 positive Bcx, 92 were matched, and 48 met inclusion criteria. *Enterobacterales* was found in 28 (58.3%), whereas PsA was found in 5 (10.4%) [Figure 1]. Gram negative bacteremia was more common than gram positive [Figure 2]. The most common source of bacteremia was intra-abdominal (n=17, 35.4%), followed by line-associated infection (n=9, 18.8%) [Figure 3]. All 48 cases were started on empiric anti-PsA abx; 31 (72.1%) were continued on anti-PsA abx for an average of 11 days [IQR 5-14] despite no PsA in Bcx. 30-day all-cause mortality was not significantly different between those who continued on anti-PsA abx versus those in whom anti-PsA abx was stopped when PsA was not isolated [5/31 versus 3/13, p=0.5]. All cases of PsA bacteremia had at least one risk factor for nosocomial acquisition, e.g. hospitalization and/or abx exposure ≤ 90 days.

**Figure 1:**
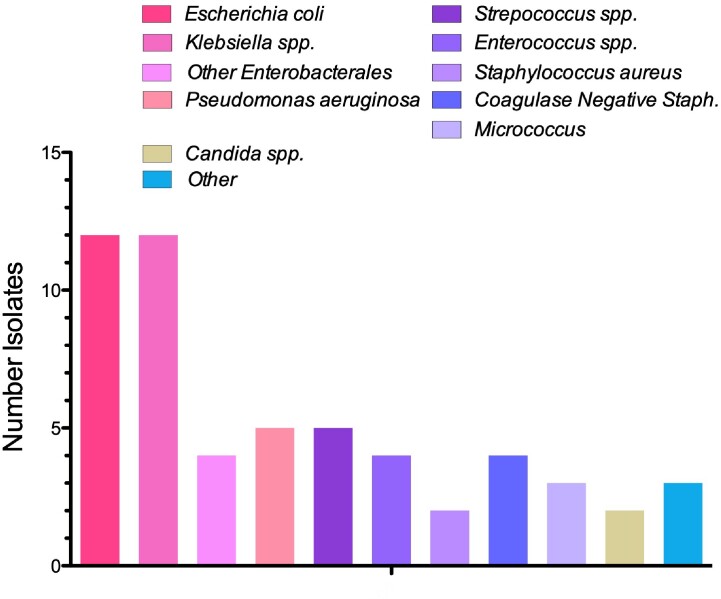
Frequency of Isolated Organisms

**Figure 2:**
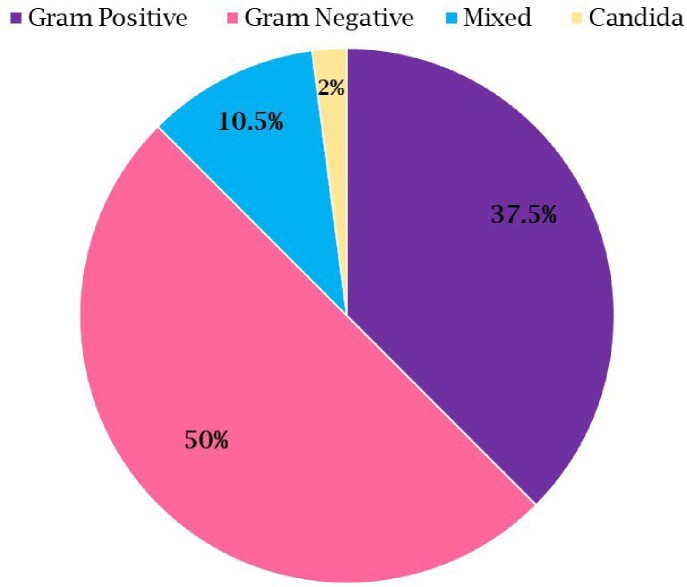
Breakdown of Organism Classifications

**Figure 3:**
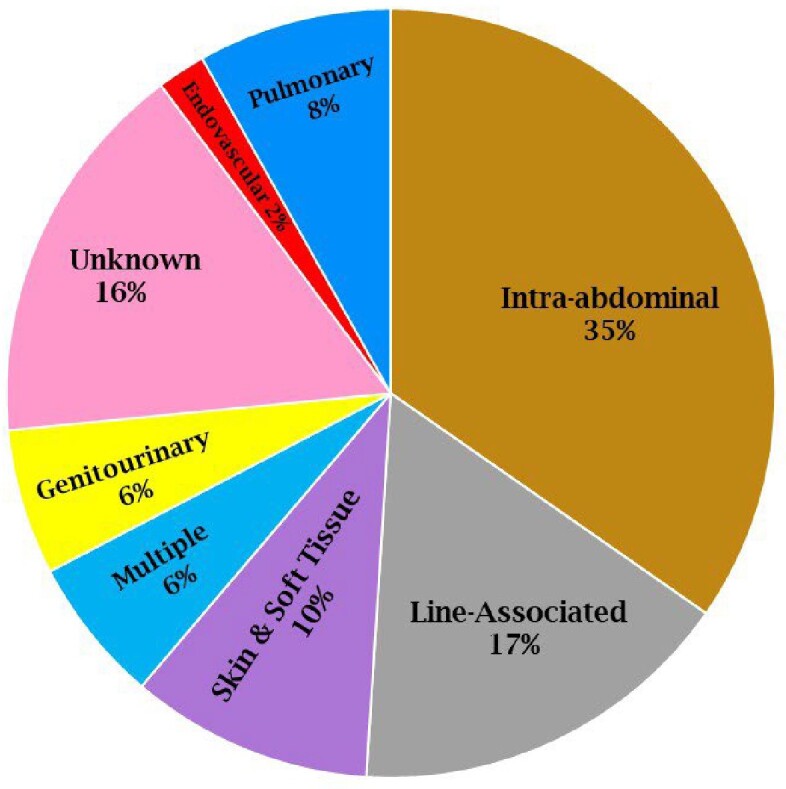
Sources of Bacteremia

**Conclusion:**

The most common organism causing bacteremia in FN was *Enterobacterales*. The prevalence of PsA bacteremia was low, yet most were continued on anti-PsA abx despite Bcx showing a different organism. Continuation of anti-PsA abx did not make a difference in all-cause 30-day mortality when PsA was not isolated. Even when neutropenic, the risk factors for PsA bacteremia was the same as that for other patients; thus, as an abx de-escalation strategy, patients can be risk stratified. Further stewardship efforts can be focused on de-escalating anti-PsA abx in FN when PsA is not isolated.

**Disclosures:**

**All Authors**: No reported disclosures

